# The RNA-binding protein PCBP1 modulates transcription by recruiting the G-quadruplex-specific helicase DHX9

**DOI:** 10.1016/j.jbc.2024.107830

**Published:** 2024-09-27

**Authors:** Joseph A.Q. Karam, Cécile Fréreux, Bidyut K. Mohanty, Annamarie C. Dalton, Toros A. Dincman, Viswanathan Palanisamy, Breege V. Howley, Philip H. Howe

**Affiliations:** 1 Department of Biochemistry and Molecular Biology, College of Medicine, Medical University of South Carolina, Charleston, South Carolina, USA; 2 Department of Cell Biology and Physiology, Edward Via College of Osteopathic Medicine, Spartanburg, South Carolina, USA; 3 Division of Hematology and Oncology, Department of Medicine, Medical University of South Carolina, Charleston, South Carolina, USA; 4 Division of Molecular Medicine, Department of Internal Medicine, UNM Comprehensive Cancer Center, University of New Mexico, Albuquerque, New Mexico, USA; 5 Hollings Cancer Center, Medical University of South Carolina, Charleston, South Carolina, USA

**Keywords:** PCBP1, G-quadruplex, transcription, DHX9, RNA/DNA hybrid

## Abstract

PCBP1, polycytosine (poly(C)) binding protein 1, an RNA and single-stranded DNA (ssDNA) binding protein, binds poly(C) DNA tracts but it remains unclear whether its ability to bind ssDNA contributes to transcriptional regulation. Here, we report that PCBP1’s DNA binding sites are enriched at transcription start sites and that by binding to promoter regions, PCBP1 regulates transcription in addition to splicing and translation. At PCBP1 target genes, we show that PCBP1 interacts with several RNA/DNA hybrid (R-loop) associated G-quadruplex resolving helicases. Furthermore, we find that PCBP1 interacts with RNA Helicase A (DHX9) to modulate transcription by regulating DHX9 accumulation and activity. PCBP1 depletion leads to defects in R-loop processing and dysregulation of transcription of PCBP1 target genes. PCBP1’s high sequence specificity and interaction with helicases suggest that its mechanism in transcription involves guiding helicases to specific loci during transcription, thereby modulating their activity.

Double-stranded DNA is typically found in its canonical form known as B-DNA. The formation of non-canonical DNA structures is influenced by sequence and environmental factors that can alter a myriad of processes occurring in DNA (1). Transcription, replication, and DNA repair, processes essential to the survival of both normal and cancerous cells, depend on the action of helicase proteins that transiently create single-stranded DNA (ssDNA). Alterations in the function of ssDNA binding proteins can lead to genome instability (2) which drives tumorigenesis and underlies the six hallmarks of cancer by promoting the heterogeneity of cancers (3). Structures such as G-quadruplexes (G4s), intercalated motifs (i-motifs), and hairpins form in both ssDNA and RNA. These structures are critical for the processing of nucleic acids. RNA:DNA hybrids (R-loops), highly stable three-stranded structures that form during transcription and DNA damage, provide further opportunity, in the form of the displaced ssDNA strand, for RNA binding proteins to bind to DNA.

Four protein domains have been characterized to interact with ssDNA: oligonucleotide/oligosaccharide-binding (OB)-fold, RNA recognition motifs (RRMs), whirly domains, and hnRNP K homology (KH) domains. Together, this subset of the proteome tightly regulates processes involving RNA and ssDNA to protect the genome from damage (4). The KH domain is unique among the four ssDNA binding domains because it binds with sequence and structure specificity. PCBP1, polycytosine (poly(C)) binding protein 1, also known as hnRNP E, and hnRNP K, two prominent members of the KH domain family, bind poly(C) tracts in DNA and RNA and have been shown to regulate DNA secondary structure homeostasis by binding to i-motif forming poly(C) sequences in gene promoters and telomeric DNA (5, 6). Our previous findings show that G4 structures, found complementary to i-motif forming sequences, are upregulated upon depletion of PCBP1 (6). In addition to PCBP1’s i-motif binding and subsequent G4 regulation, our studies suggest that the cytokine transforming growth factor beta (TGF*β*) induces epithelial to mesenchymal transition (EMT) by phosphorylating and releasing PCBP1 from a structurally conserved hairpin secondary structure (known as beta-activated translational BAT elements) found at splice sites and in the 3′-UTR of pro-EMT mRNAs thereby influencing both alternative splicing and translation, respectively (7, 8, 9, 10).

PCBP1 is ubiquitously expressed, highly conserved in eukaryotes, and is primarily localized to the nucleus (11). Depletion of PCBP1 is associated with EMT, tumor progression, increased metastatic potential, and increased cancer cell stemness (12). These cellular processes depend on both transcriptional and post-transcriptional regulatory mechanisms. Genomic instability and transcriptional reprogramming synergistically drive both EMT and the evasive heterogeneity of cancer, in turn causing the major hurdle in clinical oncology, therapeutic resistance (13). There are a few examples of transcriptional regulation by PCBP1. Most notably, it promotes the transcription of human and mouse mu opioid receptor genes (MOR). A complex consisting of all five PCBPs binds to the proximal promoter poly(C) tract of human MOR. Unlike transcription factors, the PCBPs bind ssDNA, formed in the proximal promoter, to regulate MOR activity (14, 15, 16). On a global level, PCBP1 is required for maternally induced transcriptional silencing during mouse oocyte development (17), possibly contributing to the embryonic lethality of PCBP1 KO in mice and pointing to the evolutionary importance of PCBP1.

Like PCBPs, the DExD-box (DDX) and DExH-box (DHX) helicases are critical cellular modulators, particularly in RNA and ssDNA metabolism and regulation (18). These highly conserved proteins, named after their Asp-Glu-X-Asp/His residue motif, are ATP-dependent helicases with variable expression among different cell types and in various early development stages. Like PCBP1, these helicases are multilevel regulators of genetic information encompassing roles in transcriptional regulation, splicing, mRNA stability, translational regulation, and mRNA transport (18). Recent interest in the DDX and DHX proteins has been spurred by their aberrant expression in cancer and their impact on R-loop structures. R-loops play vital roles in regulating gene expression by modulating multiple steps of transcription (19). G4 structure formation in displaced, non-template ssDNA has been shown to induce R-loops and promote transcription (20). RNA Helicase A, also known as DHX9, is known to regulate R-loop homeostasis by resolving G4 structures on the displaced non-template ssDNA (21). Herein, we show that PCBP1 binds to poly(C) rich sequences complementary to known G4 forming sequences found near transcription start sites (TSSs). At these promoter regions, present in numerous genes, PCBP1 interacts with DHX9 to regulate transcriptional activity. Loss of PCBP1 leads to G-quadruplex accumulation and R-loop formation at PCBP1 binding loci, resulting in transcriptional defects and genome instability.

## Results

### PCBP1 binds promoter polycytosine tracts

**Figure 1:**
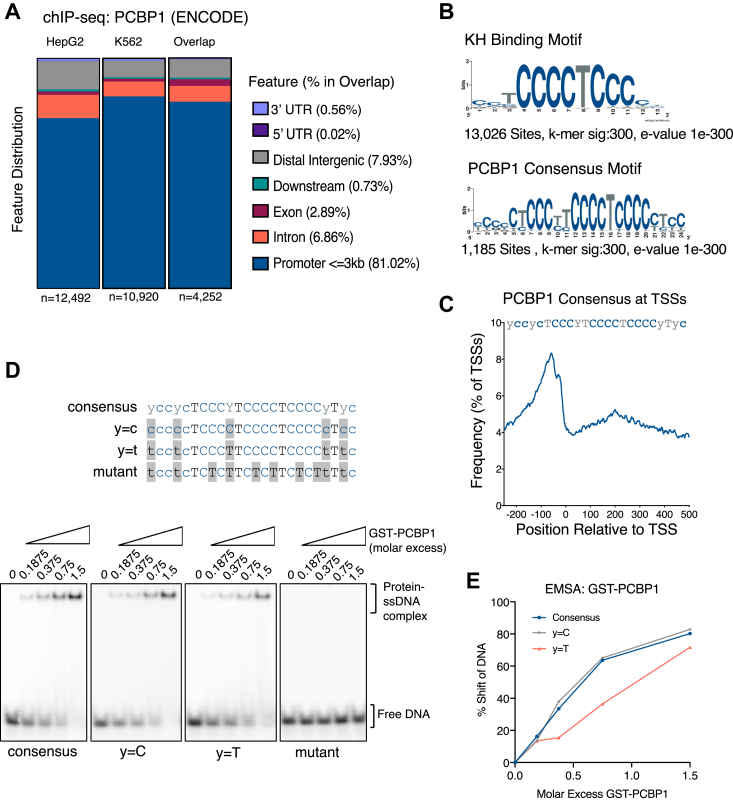
**PCBP1 binds promoter polycytosine tracts.***A*, feature distribution plots of PCBP1-enriched peaks in HepG2 (n = 12,492 peaks), K562 (n = 10,920 peaks), and peaks found in both datasets (“overlap”, n = 4252 peaks), show that greater than 80% of PCBP1 ChIP binding sites are within 3 kb of TSSs. *B*, *de novo* motif analysis using RSAT reveals poly(C) motifs as highly enriched motifs from PCBP1-enriched peaks in both HepG2 and K562 cell lines. *C*, the PCBP1 consensus motif “yccyTCCCYTCCCCTCCCCyTyc” is found 50-100 nts upstream of ∼8% of human TSSs. *D*, electromobility shift assays (EMSAs) using GST-PCBP1 to test the PCBP1 affinity for the consensus motif identified by ChIP. Four DNA probes were used, the consensus probe containing a random mixture of pyrimidines (C/T) at “y” positions, a probe with cytosines at “y = C” positions, a probe with thymidines “y = T”, and a mutant with no polycytosine tracts as a negative control. *E*, quantification of PCBP1’s binding to probes shown in (*D*), these graphs represent triplicate experiments and were used to calculate a 50% shift in radiolabeled DNA.

PCBP1 binds single-stranded poly(C) tracts with high specificity (22). We previously showed that depletion of PCBP1 leads to DNA damage and that its binding to DNA increases upon induction of DNA damage (6), suggesting that PCBP1 binding to poly(C) tracts could regulate transcription. To determine whether PCBP1 is directly involved in transcription, we utilized PCBP1 ChIP-sequencing datasets from the ENCODE database for both HepG2 (human hepatocellular carcinoma) and K562 (human myelogenous leukemia) cells (23). We defined the genomic distribution of PCBP1’s binding sites using chromatin immunoprecipitation (ChIP) ChIPseeker (24) and our analysis revealed 4252 PCBP1-ChIP binding sites commonly present in both HepG2 and K562 cells, 81.02% of the sequences were observed in promoter regions (Figs. 1*A* and S1*A*). Furthermore, in genes where PCBP1 binding occurs at the promoter, peak intersection analysis revealed that these genes were also more likely to exhibit PCBP1 binding at intronic regions. This co-occurrence of PCBP1 binding at both promoters and introns (Fig. S1*B*) suggests that PCBP1 may be involved in regulating multiple steps of transcription of target genes.

To define PCBP1 bound *cis*-elements, we used RSAT peak-motif and observed that PCBP1 bound sequences are enriched with several poly(C) tracts. The canonical KH-domain binding motif, “TCCCT”, was noted in 13,026 sites within the sequences of PCBP1 bound regions found in K562 and HepG2 cells (Fig. 1*B*). Previously, we showed that multiple poly(C) repeats increase PCBP1 binding affinity to nucleic acids (6). Thus, we decided to focus on a consensus motif, “YCCYCTCCCYTCCCTCCCCyTyc,” which is present in 1185 PCBP1-ChIP sequences (Fig. 1*B*). Using the signal search analysis (SSA) servers OProf package (25), we found that the identified consensus poly(C) tract is enriched −50 nt upstream of the TSSs in 8% of annotated human TSSs (Fig. 1*C*). Interestingly, when assessing the distribution of the poly(C) consensus motif within PCBP1-ChIP peak sequences, we observed an atypical bimodal enrichment of the motif relative to ChIP peak centers. When performing ChIP-seq of a transcription factor, its binding motif is expected to occur at the center of a distribution plot (26). Several other motifs were observed in our analysis, notably the distribution of the transcription binding motifs for ELF and KLF4, which were enriched in PCBP1-ChIP binding sites, exhibit randomness across the ChIP-seq sites, further supporting the concept that PCBP1 exhibits ssDNA binding and does not serve as a transcription factor (Figs. 1*C* and S1*D*).

To determine the ssDNA binding activity of PCBP1, we tested the binding affinity of the consensus motif in Figure 1*B* using GST-tagged PCBP1 and ^32^P-labeled DNA oligos by electromobility shift assays (EMSA; Fig. 1*D*). We used three versions of the motif: the consensus containing an equal mixture of the pyrimidines (C&T) at “Y” positions; a version representing the motif with all Y nucleotides as cytosines; and one where all Y nucleotides in the consensus are Ts. The “y = T” version of the consensus sequence exhibits slightly weaker binding than the randomized consensus and “y = C” (Fig. 1*D*). A 50% shift in substrate occurred at 0.775 and 0.745 M excess of protein over DNA for the consensus and y = C DNA oligos, while a 1.042 M excess of protein over DNA was required for a 50% shift in the y = T oligo (Fig. 1*E*). In previous work, we performed EMSAs on several DNA and RNA substrates that required a molar excess ranging from 4.4 fold for a 28 nucleotide telomeric DNA C-strand oligo to an 11 fold molar excess of GST-PCBP1 for a 50% shift in BAT-element RNA oligos (6, 27). This suggests that the consensus sequence identified in promoters through ChIP-seq analysis has a high affinity for PCBP1 and is enriched at transcriptionally functional loci.

### Regulation of transcription by PCBP1 through DNA binding

**Figure 2:**
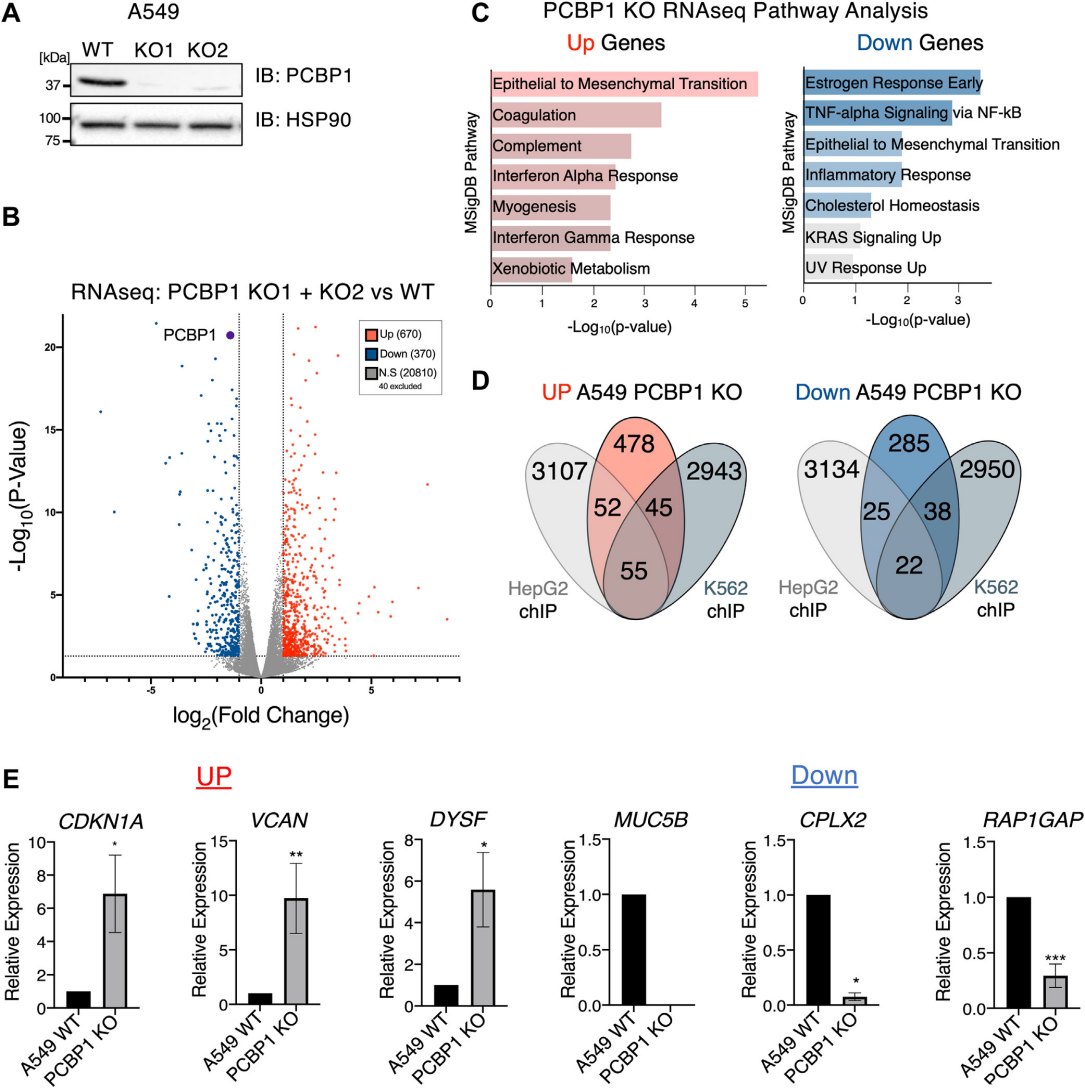
**PCBP1 binds DNA to regulate transcription.***A*, immunoblot of two A549 PCBP1 KO clones. *B*, volcano plot of PCBP1 KO RNA-seq data, differentially expressed genes that are significantly (−Log_10_ of *p*-value greater than, greater than 2-fold change) upregulated (*red*, n = 670) and downregulated (*blue*, n = 370). *C*, gene set enrichment using msigDB hallmark pathway gene sets of up and down differentially expressed genes. *D*, Venn diagrams showing overlap of differentially expressed genes and PCBP1-enrichment from ChIP-seq. 152/670 of upregulated and 85/370 of downregulated genes have promoter region PCBP1 enrichment. *E*, RT-qPCR validation of differentially expressed genes with PCBP1-enriched DNA identified by ChIP-seq. A single asterisk (∗) signifies a *p*-value less than 0.05, (∗∗) signifies *p*-value less than 0.01, and (∗∗∗) signifies *p*-value less than 0.001.

To determine the functional role of PCBP1 at promoter regions, we generated PCBP1 CRISPR KO A549 (human lung adenocarcinoma) cells (Fig. 2*A*). We performed RNA-sequencing of two A549 PCBP1 KO cell lines and found 630 genes that are upregulated and 370 genes that are significantly downregulated greater than two-fold between the knockout (KO) and wild-type (WT) samples (Fig. 2*B*). We used Gene Set Enrichment Analysis (GSEA) and the MSigDB hallmark to run pathway analysis on differentially expressed genes. Amongst upregulated genes, we observed high enrichment of epithelial to mesenchymal transition pathway genes (Fig. 2*C*), reinforcing what was previously known about the depletion of PCBP1 (8, 28, 29). We looked at the genes that have PCBP1 binding sites identified through our analysis of the ENCODE PCBP1 ChIP-seq data to determine if transcriptional regulation correlated with binding to promoters. We found, in A549 cells, that 152 targets out of 630 of the genes upregulated by PCBP1 KO and 85 out of 370 of the downregulated genes were found to have PCBP1 bound promoters in the PCBP1 ChIP-seq data from HepG2 and K562 cell lines (Fig. 2*D*). We confirmed the regulation of three upregulated (*CDKN1A*, *VCAN*, *DYSF*) and three downregulated (MUC5B, *CPLX2*, *RAP1GAP*) genes that have promoter ChIP binding sites in the PCBP1 ChIP-seq data by qRT-PCR (Fig. 2*E*). Altogether, the presence of poly(C) locations in the promoters and the conserved and widespread expression of PCBP1 across cell types suggest a conserved role for PCBP1 in transcriptional regulation through promoter binding.

### Evolutionarily conserved regulation of *CDKN1A* by promoter DNA binding

**Figure 3:**
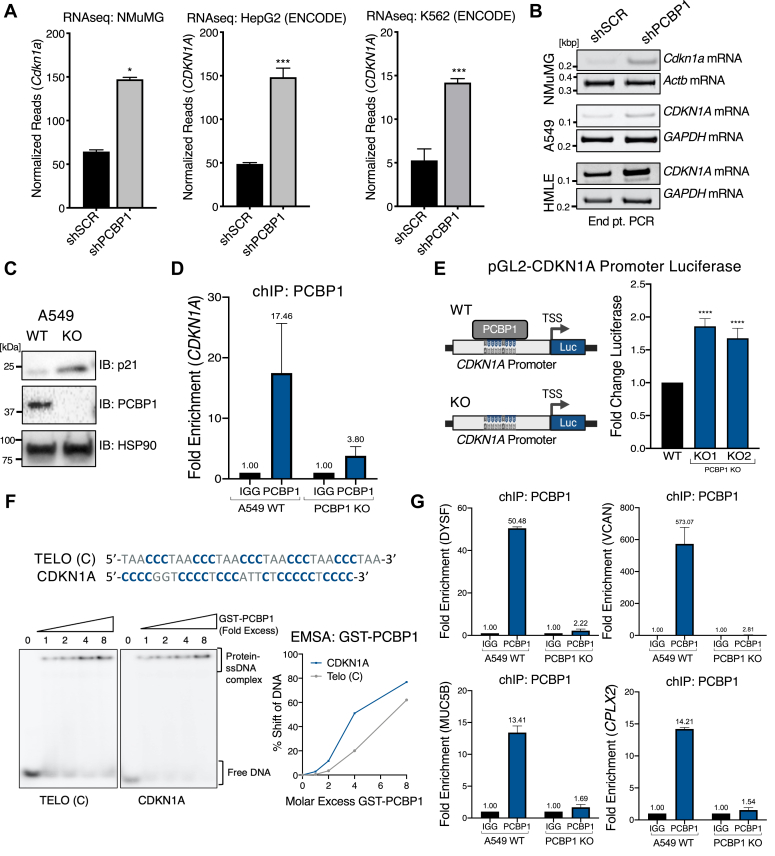
**PCBP1 regulates transcription of *CDKN1A*.***A*, *CDKN1A* transcript levels from NMuMG, HepG2, and K562 PCBP1 knockdown RNA-seq datasets. *B*, endpoint PCR products showing increased *CDKN1A* in NMuMG, A549, and HMLE cell lines in WT and PCBP1 knockdown cell lines. *C*, immunoblot of p21 in A549 WT and PCBP1 knockout cells. *D*, PCBP1ChIP-qPCR showing using probes for the *CDKN1A* promoter, in WT compared to PCBP1 knockout cells as a negative control. *E*, *CDKN1A* promoter luciferase assay to measure effect of PCBP1 knockout on luciferase expression. *F*, EMSA to test GST-PCBP1 affinity to poly(C) motif found in *CDKN1A* promoter DNA, Telomeric C-strand DNA was used as a control. Experiments were performed in triplicate and a representative graph of PCBP1 binding used to calculate a 50% shift is shown. *G*, PCBP1 ChIP-PCR of promoters from A549 WT and PCBP1 knockout (negative control) lysates, reveals PCBP1-enrichment at *DYSF*, *VCAN*, *MUC5B*, and *CPLX2* promoters. A single asterisk (∗) signifies a *p*-value less than 0.05, (∗∗∗∗) signifies *p*-value less than 0.01, and (∗∗∗) signifies *p*-value less than 0.001.

Previous work on PCBP1 has indicated the conservation of its structure, expression, and mechanisms across eukaryotes (30, 31), leading us to question whether differentially expressed genes are conserved upon PCBP1 depletion across species. We chose to examine the cyclin-dependent kinase inhibitor 1A (*CDKN1A*) gene that encodes the p21 tumor suppressor protein (32) for several reasons. First, we show that *CDKN1A* is upregulated upon PCBP1 depletion in A549 cells (A549 PCBP1 KO) (Fig. 2*E*). In addition, in non-transformed mouse mammary gland epithelial (NMuMG) cells, we previously demonstrated by RNA-seq analysis that silencing of PCBP1 led to an upregulation of *CDKN1A* (33) and confirmed its upregulation in HepG2 and K562 cells in RNA-seq analysis of the ENCODE database (Fig. 3*A*). We further confirmed *CDKN1A* mRNA upregulation in A549, human mammary epithelial cells (HMLE) cells, and mouse NMuMG cells upon shRNA-mediated knockdown of PCBP1 by RT-PCR (Fig. 3*B*). We verified p21 upregulation at the protein level by immunoblotting upon PCBP1 knockout in A549 cells (Fig. 3*C*) in HeLa cells (Fig. S2*A*), and by immunofluorescence (Fig. S2*B*). The upregulation of p21 in HeLa cells upon PCBP1 KO suggests p53-independent regulation of *CDKN1A* transcription as HeLa S3 cells are p53-deficient (32). The HepG2 and K562 ChIP-seq datasets confirmed that PCBP1 binds the *CDKN1A* promoter. PCBP1 binding sites from both cell lines and their overlap are found in Table S1. We tested whether this binding is conserved in A549 cells and observed significant enrichment of *CDKN1A* promoter DNA upon ChIP using PCBP1 antibody (Fig. 3*D*). To determine if PCBP1’s regulation of *CDKN1A* expression is mediated through binding to its promoter region, we used a proximal promoter luciferase assay containing only the promoter upstream of the TSS. We observed an upregulation in transcriptional activity upon PCBP1 knockout (Fig. 3*E*), leading us to ask whether the DNA binding consensus motif identified in Figure 1 was also present in the *CDKN1A* promoter sites present in PCBP1 ChIP seq data. We found several poly(C) motifs in *CDKN1A*’s promoter and tested their affinity for GST-PCBP1 binding by EMSA. We observed a 50% shift in the *CDKN1A* poly(C) tract oligo we tested occurred at a GST-PCBP1 molar excess of 5.03 compared to an excess of 6.92 for the telomere C strand oligo (Fig. 3*F*).

In addition to *CDKN1A,* we identified several other conserved differentially expressed genes by comparing HepG2 PCBP1 knockdown RNA-seq and PCBP1 ChIP-seq data to NMuMG PCBP1 knockdown gene expression data established in our laboratory (GSE94637). RNA-seq expression levels for *CSF1* (upregulated), *BNIP3* (downregulated), and *NDRG1* (downregulated) showed a strong correlation in both HepG2 and NMuMG cell lines (Fig. S2*C*). We confirmed their levels by PCR in NMuMG and tested levels in A549 cells stably expressing shRNA targeting PCBP1 (Fig. S2*D*). We used PCBP1 ChIP/PCR, to show that PCBP1 binds to the *CDKN1A*, *BNIP3*, *CSF1*, and *NDRG1*, proximal promoters in A549 cells (Fig. S2*E*). These genes, apart from *CDKN1A*, did not rank amongst the most differentially expressed genes in our A549 PCBP1 KO dataset, so we decided to study PCBP1’s transcriptional role further on *DYSF*, *VCAN*, *MUC5B*, and *CPLX2*, genes highly modulated by PCBP1 in A549 cells. By performing ChIP in WT and PCBP1 KO A549 cells using PCBP1 antibody, we observed significant enrichment of promoter DNA from these four genes in WT cells (Fig. 3*G*). Thus, the subset of genes regulated by PCBP1 and the levels at which they are expressed may vary in given cell types. However, the discovery of cross-species conserved regulation motivated us to understand the specific conditions under which PCBP1 binds to ssDNA at the promoter region.

### Silencing PCBP1 leads to G-quadruplex accumulation in promoter regions

**Figure 4:**
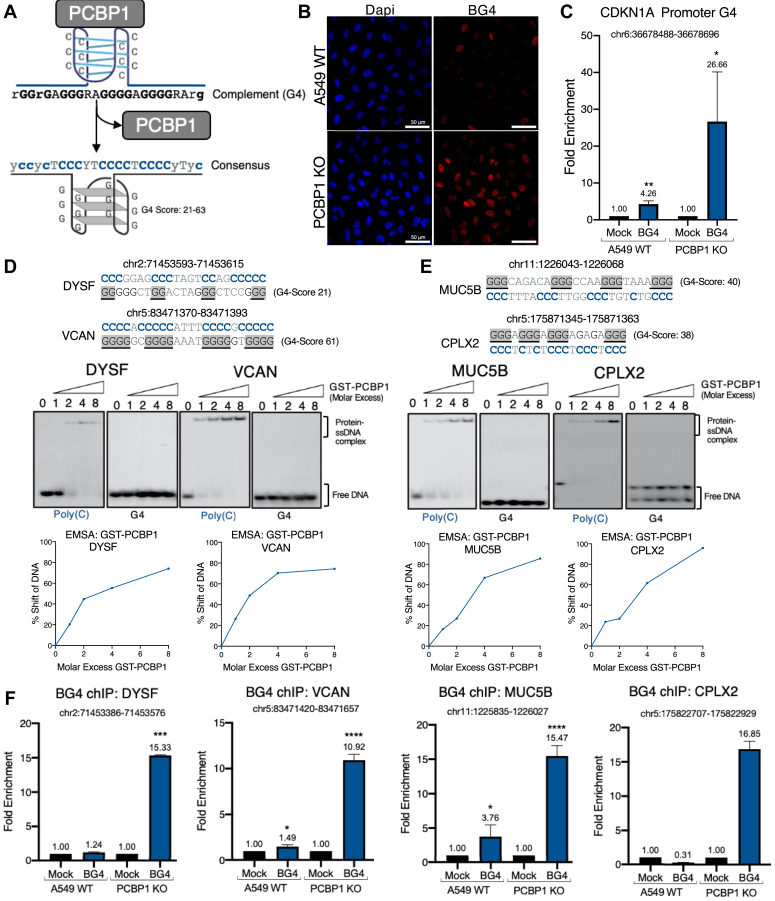
**Silencing PCBP1 leads to promoter G4 accumulation.***A*, model showing G4 formation upon PCBP1 depletion and QGRS score of G4-formed by sequence complementary to the PCBP1 consensus motif. *B*, immunofluorescence of A549 WT and PCBP1 KO cells stained with DAPI and BG4 (anti-G4 structure) antibody. *C*, BG4 ChIP-PCR of *CDKN1A* promoter DNA from A549 WT and PCBP1 KO nuclear lysates. *D*, GST-PCBP1 EMSAs of poly(C) sites in the promoters of upregulated differentially expressed genes, *DYSF* and *VCAN*. Representative graphs used to calculate 50% gel shifts for each probe are shown. *E*, GST-PCBP1 EMSAs of poly(C) sites in the promoters of downregulated differentially expressed genes, *MUC5B* and *CPLX2*. Representative graphs used to calculate 50% gel shifts for each probe are shown. *F*, BG4 ChIP-PCR of promoters of *DYSF*, *VCAN*, *MUC5B*, and *CPLX2* from A549 WT and PCBP1 KO nuclear lysates. A single asterisk (∗) signifies a *p*-value less than 0.05, (∗∗) signifies *p*-value less than 0.01, (∗∗∗) signifies *p*-value less than 0.001, and (∗∗∗∗) signifies a *p*-value less than 0.0001.

We previously observed that PCBP1 depletion by shRNAs in mouse and human cells leads to loss of i-motifs, structures formed from poly(C) sequences, and upregulation of G4 structures across the genome (6). I-motif and G4 structures can occur at the same loci on opposite strands, and the forms are thought to be mutually exclusive due to steric hindrance (34). This led us to use QGRS mapper (35) to assess the predicted G4 score of the strand complementary to the poly(C) site in the *CDKN1A* proximal promoter that we tested by EMSA and the PCBP1 consensus motif identified through analysis of PCBP1 ChIP-seq data. We found that both sequences complement potential G-quadruplex (G4) forming sequences. For the ChIP-seq consensus motif, QGRS gave scores ranging from 21 for the weakest binding “y = T” form of the consensus to 63 for “y = C”. Sequences with a QGRS score greater than 20 are considered highly probable G4-forming sequences. A schematic summary of the data is shown (Fig. 4*A*). We verified that G-quadruplex structures are upregulated upon PCBP1 depletion using immunofluorescence and staining with the BG4 antibody in A549 PCBP1 KO cells (Fig. 4*B*). The data, showing increased levels of BG4 staining upon PCBP1 depletion, suggest that PCBP1 suppresses G4 structure formation by binding at poly(C) sequences present in the complementary strand. To confirm whether G4-sequences are indeed critical for gene expression, we used the BG4 antibody to ChIP the PCBP1 binding site identified through PCBP1 ChIP-seq at the *CDKN1A* promoter and found a significant enrichment of the region in PCBP1 KO cells (Fig. 4*C*). This finding is confirmed by using QGRS Mapper to identify G-quadruplex sequences within PCBP1 binding sites identified by HepG2 and K562 PCBP1 ChIP-seq (Fig. 1*A*). To confirm the binding of PCBP1 to G4 complementary strand poly(C) sequences we chose the highest-scoring potential G4 sequences and tested whether the complementary sequences bound to PCBP1 (Fig. S3*A*). We used this strategy to test for G4s in the promoters of the most significant differentially expressed genes in our A549 PCBP1 RNA-seq dataset and found that in upregulated genes, the highest scoring promoter G4 is found on the non-template strand in 13/15 of the top upregulated genes (Fig. S3*B*). We used GST-PCBP1 to perform EMSAs, which demonstrated PCBP1’s *in vitro* binding to sequences within the promoters of upregulated (*DYSF* & *VCAN*) and downregulated (*MUC5B* & *CPLX2*) DNA sequences (Fig. 4, *D* and *E*). We performed ChIP analysis, using BG4 antibodies, to test if these regions formed G4 structures and observed that PCBP1 KO led to G4 DNA enrichment (Fig. 4*F*). As a negative control, we tested enrichment by the BG4 antibody at loci in *DYSF*, *VCAN*, *MUC5B*, and *CPLX2* not predicted to form G4s, and none exhibited enrichment (Fig. S3*C*); a five-fold enrichment relative to input by chIP-qPCR is a metric that signifies G4-positive loci (36). We next used two small molecules that stabilize G4 structures, BRACO-19 (37) and Phen-DC3 (38), to mimic the effect of PCBP1 knockout on gene expression. We found that BRACO-19 and Phen-DC3 treatment of WT A549 cells results in the upregulation of *DYSF* and *CDKN1A* mRNA (Fig. S3*D*). For the downregulated genes, we observed reduced expression of *CPLX2* and MUC5B only under treatment with Phen-DC3 and BRACO-19, respectively (Fig. S3*E*). Collectively the data support the hypothesis that PCBP1’s regulation of gene expression is associated with its binding to ssDNA complementary to G4 structures and its contribution to the resolution of G4 structures.

### PCBP1-DHX9 interactions occur at promoter regions

**Figure 5:**
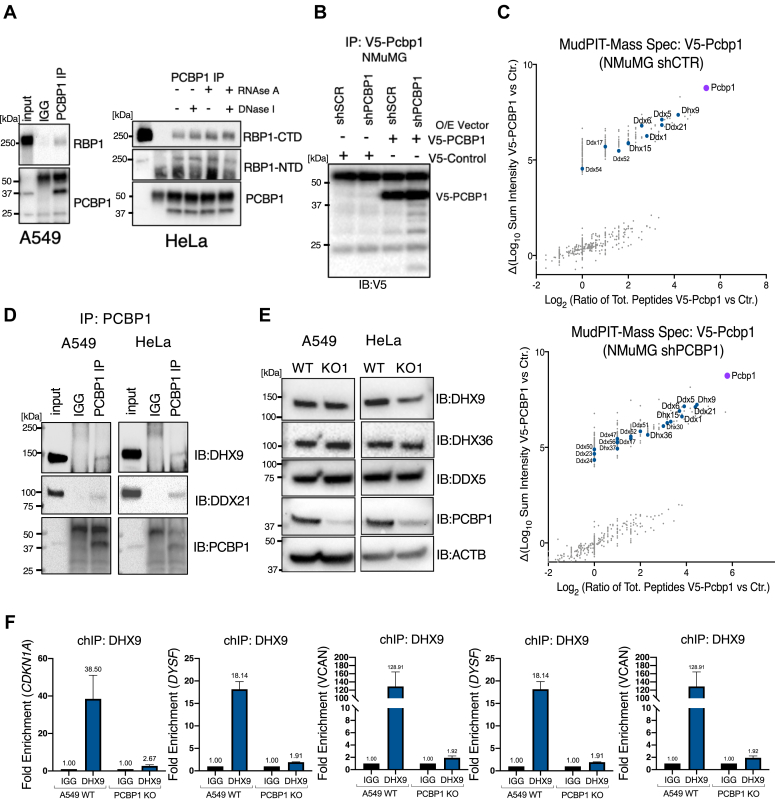
**PCBP1 interacts with DHX9 at promoters.***A*, co-IP of PCBP1 to test interaction with RNA Pol II. Immunoblotting of PCBP1 and RBP1, the major DNA binding subunit of RNA Pol II in A549 and HeLa cells. HeLa lysates were treated with RNAse A, DNAse I, or both to test the requirements of DNA and RNA for PCBP1-Pol II interaction (*right panel*). *B* and *C*, co-IP and mass spectrometry to identify PCBP1 interactors in NMuMG cells transfected with V5-control and V5-PCBP1 vectors. Plots show enriched PCBP1 protein interactors from V5-Pcbp1 transfected NMuMG shSCR and shPCBP1 cells. *D*, co-IP of PCBP1 to validate interaction with DHX9 and DDX21 in human A549 and HeLa cells. *E*, immunoblots showing expression of RNA and DNA G4 resolving helicases DHX9, DHX39, and DDX5 from A549 and HeLa WT cells and their PCBP1 KO derivatives. *F*, DHX9 ChIP-PCR from A549 WT and PCBP1 KO cells to test DHX9-enrichment at *CDKN1A*, *DYSF*, *VCAN*, *MUC5B*, and *CPLX2* promoter DNA.

G4 forming sequences are found throughout the genome and our data demonstrates that PCBP1 preferentially binds to promoter DNA near TSSs where G4 structure formation can both negatively and positively regulate RNA Pol II progression (39). This led us to investigate whether PCBP1 binds to or is associated with the RNA Polymerase II complex. Using co-immunoprecipitation (co-IP), we show that RNA pol II subunit A (RBP1), the major DNA binding subunit of RNA Pol II, interacts with PCBP1 in A549 and HeLa cells (Fig. 5*A*; left panel). Furthermore, by treating with RNAse and DNAse, we show that the interaction is independent of DNA or RNA (Fig. 5*A*; right panel). To gain further support and insight into PCBP1’s role as a multifunctional co-transcriptional regulator we probed its protein interactome. We transiently overexpressed V5-tagged PCBP1 in control NMuMG (shCTR) and NMuMG attenuated for PCBP1 by shRNA-mediated silencing (shPCBP1) and V5-co-precipitates were analyzed by mass spectrometry (Fig. 5*B*). When examining the interactors, we observed an enrichment of members of the DHX and DDX proteins. In humans, 36 DDX proteins and 14 DHX proteins have been characterized (40). Of these proteins, 16/36 DDX proteins and 5/14 DHX proteins were identified by our mass spectrometry analysis as PCBP1 interactors in NMuMG cells (Fig. 5*C*). We next used co-IP to validate PCBP1’s interaction with the two most enriched helicases, DHX9 and DDX21, in human A549 and HeLa cells (Fig. 5*D*). To rule out the possibility that PCBP1 regulates the expression of these DNA/RNA G4-resolving helicases post-transcriptionally, we verified that their protein levels remain stable upon PCBP1 knockout in A549 and HeLa cells (Fig. 5*E*).

As DDX21 is only known to resolve RNA G4 structures (41, 42) we decided to focus on helicases that are known to resolve G4 structures in both DNA and RNA, DHX9, DHX36, and DDX5 (43, 44, 45). We chose to further investigate DHX9 as it is the most enriched PCBP1 interacting helicase in our mass spectrometry data (Fig. 5*C*), acts on both RNA and DNA and is a well-characterized transcriptional regulator (46, 47). In addition, DHX9 has been shown to modulate RNA/DNA hybrid (R-loop) levels through its ability to resolve G4 structures and several R-loop interactome studies have characterized the enrichment of KH domain-containing RNA binding proteins and DHX helicases, including PCBP1 and DHX9 (48, 49). To determine whether DHX9 acts on PCBP1 target gene promoters, we employed DHX9 ChIP followed by PCR to assess DHX9’s binding at the promoters of *CDKN1A*, *DYSF*, *VCAN*, *MUC5B*, and *CPLX2.* We found that DHX9-ChIP enriches *CDKN1A* DNA and promoter DNA from our other target genes and that DHX9 enrichment is lost upon PCBP1 knockout (Fig. 5*F*). These observations indicate that DHX9’s recruitment to loci is coordinated by PCBP1, and that epistatic interaction between DHX9 and PCBP1 is required for a subset of transcriptionally active genes.

### PCBP1 depletion increases R-loops by stabilizing G4 structures

**Figure 6:**
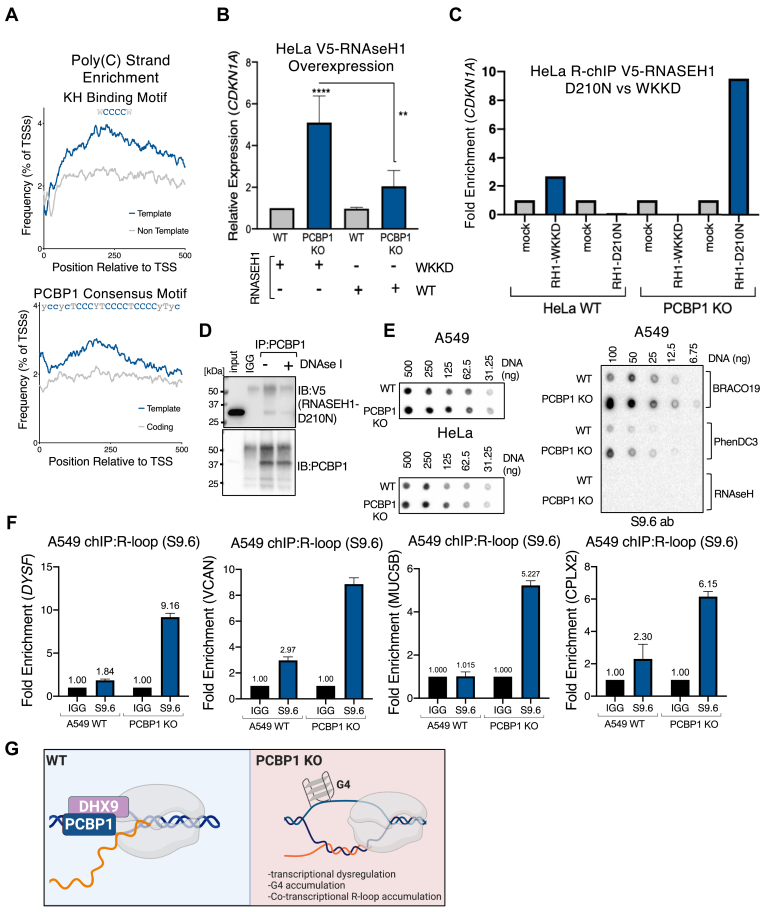
**PCBP1 depletion increases promoter R-loops.***A*, frequency plots of KH binding motif and PCBP1 consensus motif at position 0 to 500 relative to human transcription start sites. *B*, RT-qPCR measuring *CDKN1A* mRNA levels in HeLa WT and PCBP1 KO cells expressing V5-RNAseH1-WKKD and V5-RNAseH1-WT. *C*, R-loop ChIP-PCR using anti-V5 antibody from nuclear lysates of HeLa cells expressing V5-tagged RNAseH1-WKKD and -D210N to measure R-loop enrichment at the *CDKN1A* promoter. *D*, co-IP of PCBP1 from HeLa cells expressing V5-RNAseH1-D210N, immunoblotting for PCBP1 and V5 signal. Cell lysates were treated with DNase to test whether PCBP1:R-loop interactions are ssDNA dependent. *E*, dot blots using the S9.6 antibody. Dot blots measure global R-loop levels in A549 and HeLa WT and PCBP1 KO cell lines and in A549 shSCR and shPCBP1 cell lines treated with G4 stabilizing drugs BRACO-19 and Phen-DC3. *F*, R-loop ChIP-PCR using the S9.6 antibody to test R-loop enrichment at promoters of *DYSF*, *VCAN*, *MUC5B*, and *CPLX2. G*, model of our findings. PCBP1 recruits DHX9 to loci by binding to polycytosine tracts. Upon PCBP1 depletion, G4s form opposite to PCBP1 binding sites due to lack of helicase activity by DHX9. PCBP1 depletion leads to transcriptional dysregulation, G4 structure stabilization, and co-transcriptional R-loop accumulation. Two asterisks (∗∗) signifies a *p*-value less than 0.01 and (∗∗∗) signifies *p*-value less than 0.001.

Strand-specific localization of G4 structures at promoter regions has been shown to regulate transcription (50). G4 structures are more likely to form on the displaced strand in R-loops as G-rich nascent RNA forms highly stable RNA-DNA hybrids with template strand DNA. Furthermore, G4 structures on the displaced non-template ssDNA, in an R-loop, can induce transcription (20); whereas, G4s on the template strand downstream of TSSs have been reported to reduce transcription by impeding RNA Pol II (51, 52, 53), ultimately leading to DNA damage (54). We found that for both the general KH-domain binding motif and the PCBP1 consensus motif, that poly(C) tracts are enriched on template strand DNA located 0 to +500 nts relative to TSSs (Fig. 6*A*). This suggests that in the absence of PCBP1 binding to poly(C) motifs, the formation of G4s on the non-template strand may be increased, thereby influencing transcriptional R-loop levels. We decided to probe R-loop levels in PCBP1-depleted cells and generated WT and PCBP1 KO HeLa cell lines stably expressing V5-tagged RNAseH vectors (RNAseH1 being responsible for the resolution of RNA: DNA hybrids in R-loops) allowing for R-loop analysis using R-ChIP (55). We overexpressed wildtype WT-RNAseH, a catalytically dead RNAseH that does not translocate to the nucleus (RNAseH-WKKD), or a catalytically dead RNAseH that binds to but does not resolve the RNA/DNA hybrid portion of R-Loops (RNAseH D210N). Expression levels of these RNAseH species in WT and PCBP1 KO HeLa cells demonstrate comparable expression levels of each form of RNAseH1 (Fig. S4*A*). We observed reduced expression of *CDKN1A* by RT-qPCR, upon PCBP1 KO in cells overexpressing both the WT RNAseH and the catalytically dead RNAseH-WKKD, indicating that resolution of an R-loop in CDKN1A by RNAseH negatively regulates its transcription (Fig. 6*B*). We next performed R-loop ChIP using V5 antibody immunoprecipitation (IP) from cellular lysates from WT and PCBP1 KO HeLa cells expressing either WKKD or the D210N RNAse1 mutants followed by qPCR analysis of the *CDKN1A* promoter. Under neither cellular condition did the WKKD RNAse1 mutant IP enrich for promoter DNA. The D210N RNAseH1 mutant IP enriched for the CDKN1A promoter in PCBP1 KO cells and not in WT types, indicating that *CDKN1A* forms R-loops that may contribute to its expression (Fig. 6*C*). Together with our DHX9-ChIP data (Fig. 5*F*), these results demonstrate that at the *CDKN1A* promoter, PCBP1 depletion, decreased DHX9 localization, and increased R-loop formation correlate with increased transcriptional activity of *CDKN1A*. To determine whether PCBP1 interacts with R-loops in the displaced ssDNA strand, we performed co-IP with PCBP1 from HeLa cell lysate overexpressing V5-RNAseH1 D210N treated ±DNAse1 to test whether degradation of the displaced ssDNA disrupts the interaction of PCBP1 with R-loop structures. We observed a slight reduction in the V5-RNAseH1-D210 co-IP signal in lysates treated with DNAse1 suggesting that PCBP1’s R-loop interaction partly depends on the displaced ssDNA (Fig. 6*D*). We used a dot blot assay using the R-loop S9.6 antibody to assess whether PCBP1 has a global impact on R-loop levels. In both A549 and HeLa cells, we observed a slight increase in R-loop levels upon PCBP1 KO (Fig. 6*E*; left panels). When we performed similar dot blot experiments, using the S9.6 antibody, with lysates from WT and PCBP1KO A549 cells treated with the G4 stabilizing drugs Phen-DC3 and BRACO-19, we found significantly higher R-loop levels in PCBP1-depleted cells (Fig. 6*E*). These results indicate that PCBP1 is involved in resolution of R-loop formation. Next, we used the S9.6 antibody to ChIP the PCBP1 binding site containing promoter regions of *DYSF*, *VCAN*, *MUC5B*, and *CPLX2* from WT and PCBP1KO A549 nuclear lysates. We observed significant R-loop enrichment for all four of our target genes upon PCBP1 KO (Fig. 6*F*), indicating that the transcriptional effect we observe, upon PCBP1 knockout, is mechanistically associated with the level of R-loop accumulation at these promoters.

In summary, our *in vitro* data and computation analyses of PCBP1 chIP-seq data show that PCBP1 is a critical protein in transcriptionally active R-loop structures. Using *CDKN1A*, *DYSF*, *VCAN*, *MUC5B*, and *CPLX2,* we have shown that PCBP1 depletion leads to dysregulated gene expression, an increase in promoter G4 formation, an associated increase in R-loop formation, and a decrease in DHX9 localization (Fig. 6*G*, model).

## Discussion

In this work, we show that PCBP1 regulates transcription by binding to ssDNA complementary to G-quadruplex structures. PCBP1’s binding to ssDNA leads to DHX9 recruitment to G4 and R-loop forming regions, thereby regulating homeostatic transcriptional activity. This work expands on our previous characterization of PCBP1 in the maintenance of genome stability by binding to ssDNA through the characterization of PCBP1’s interaction with several RNA/DNA helicases known to modulate transcription by affecting co-transcriptional R-loop activity. The ENCODE project performed ChIP-sequencing analyses on a series of RNA-binding proteins as they are known to associate with chromatin (50). RNA binding protein-chromatin interactions are generally thought to occur through interaction with transcription factors or by recruitment to chromatin *via* RNA interactions (56). Here we show that PCBP1 has a high affinity for specific ssDNA poly(C) sequences near TSSs and that its binding is RNA-independent, ultimately leading to the regulation of DNA secondary structures that impact transcription progression.

The idea that an RNA binding protein can moonlight as an ssDNA binding to regulate transcription was first suggested almost three decades ago when hnRNPK was found to bind to a poly(C) sequence in the MYC promoter to fine-tune MYC expression (57). Our investigation into PCBP1’s DNA binding began with the observation that BAT element RNA was similar in sequence to telomeric DNA. In previous work, we and others have shown that PCBP1 binds to the CCCTAA sequence at the telomeres (6, 58). Telomere 3′ ends are composed of exposed ssDNA on the G-T-rich strand, and this ssDNA can cause checkpoint activation and is critical to the function and maintenance of telomeres (59). Telomeric DNA is transcribed in one orientation to generate TERRA RNA that contains GGGUUA repeats that are implicated in R-loop formation at telomeres (60). PCBP1 has been identified as one of the highest affinity binding proteins to the C-rich strand of telomeres where it binds alongside telomeric repeat binding factor 2 (TRF2) and protection of telomeres protein 1 (POT1) (61). Although the mechanism and function of PCBP1 at telomeres are unknown, our work on DNA damage provides a hint, as telomeres are known to form i-motif structures on the C-rich strand (62). Furthermore, CDKN1A, which we show is transcriptionally repressed by PCBP1 is transcriptionally repressed by TRF2 through TRF2’s binding to a CDKN1A G-quadruplex (63). In combination with our previous study on PCBP1’s role in DNA damage and genome integrity, our current results lead us to predict that one of the mechanisms by which PCBP1 regulates genome integrity is by regulating R-loops throughout the genome including at telomeres. This is potentially through its known protein interactions with main players in telomeric secondary structure formation alongside the PCBP1-DHX9 interaction characterized in this work as DHX9 suppression is known to lead to replicative senescence (64).

The tools to study R-loops, G4s, and other phenomena that coincide with the presence of ssDNA and require housekeeping by multifunctional proteins such as PCBP1 and DHX9 have only emerged over the past few years. One mass spectrometry-based screening for R-loop interactors used two biotinylated R-loop probes to characterize R-loop interacting proteins. In that study (48), the probe containing the PCBP1 binding sequence “TCCCTCTCCCCTTCCCTCCCC” on the displaced ssDNA strand exhibited 8.8× fold higher PCBP1 binding levels, whereas other R-loop interacting proteins were found to interact with both R-loop probes suggesting more widespread roles at R-loops. This finding supports our data showing that on a global level, R-loops are modestly upregulated upon PCBP1 depletion (Fig. 6*F*). In another R-loop interactome study, 38% of R-loop interactors were found to be RNA-binding proteins, including PCBP1. In that study, DHX9 was shown to regulate transcription termination and prevent R-loop-induced DNA damage (49). Given the ubiquitous presence of KH and DDX/DHX family proteins at R-loops, their interplay and synergistic roles at R-loops and R-loop-mediated processes remain unclear. The enrichment of poly(C) sites at promoters (Fig. 1*B*) and the strand-specific enrichment of poly(C) sites on the template strand downstream of TSSs (Fig. 6*A*) were intriguing reasons to investigate PCBP1’s role in transcription by binding ssDNA. Our previous finding, that PCBP1 depletion leads to G4 upregulation (6), was confirmed here in A549 PCBP1 knockout cells (Fig. 4*B*) and at the promoters of genes whose transcription is altered upon PCBP1 depletion (Fig. 4, *C* and *F*). During transcription, G4 structures on the displaced non-template ssDNA in an R-loop are known to induce transcription (20). G4s on the template strand, downstream of TSSs, have been shown to reduce transcription by impeding RNA Pol II and stabilizing R-loops (51, 52, 53), ultimately leading to DNA damage if unresolved (54). R-loops have been shown to regulate transcriptional pausing, which occurs downstream of TSSs, as shown by R-ChIP (55). We used R-ChIP and the S9.6 antibody to show R-loop enrichment at the *CDKN1A* promoter (Fig. 6*C*) upon PCBP1 KO and subsequently an increase of G4 accumulation (Fig. 4*C*) and loss of DHX9 at the promoter (Fig. 6*F*).

DHX9 is among the best-characterized regulators of R-loop homeostasis through its ability to resolve G4s during splicing, transcription termination, DNA replication, homologous recombination, and DNA repair (21, 49, 65, 66). Several studies describe the recruitment of DHX9 to R-loop forming loci, or conversely, DHX9’s recruitment of other proteins to R-loops in the context of DNA damage and prevention of aberrant R-loop-induced damage (6). Recently, TDRD3, a protein that recognizes histone methylation marks and contains a ssDNA binding OB-fold, has been shown to recruit DHX9 to prevent R-loops at promoters and to enhance DHX9’s helicase activity (67). Most recently, DHX9 has been shown to be required for STAT1 recruitment to interferon-stimulated gene promoters, thereby regulating their transcription (68). Interestingly, our gene set enrichment analysis revealed interferon gene pathways, hinting at a PCBP1-DHX9 transcriptional co-function (Fig. 2*C*).

In our previous study on PCBP1’s role in genomic stability (6), through an *in silico* assessment of poly(C) site distribution, we found that approximately 30% of poly(C) sites are distal intergenic. This finding warrants further investigation into PCBP1’s role in DNA damage and replication (6). Recently, DHX36 which we identified as a PCBP1 interactor (Fig. 6*C*), has been shown to be required for replication of G4 DNA (69). In further support for the role of PCBP1 in replication, or a specific role in the prevention of transcription-replication conflicts, PCBP1 and its co-interactors DDX21 and DHX15 (Fig. 6, *C* and *D*) are enriched at replication forks in cells treated with the topoisomerase inhibitor camptothecin (CPT) (70). In addition to blocking replication, CPT has been well characterized to induce R-loops that lead to co-transcriptional double-strand breaks (71). Furthermore, in cells treated with CPT, DHX9 and poly(ADP)-ribose polymerase 1 (PARP1) have been shown to accumulate at CPT-induced R-loops, but reciprocal knockdown experiments showed that recruitment of DHX9 and PARP1 to R-loops is independent of one another (49). A mechanism of PARP1 recognition and binding to R-loops was reported in a recent study (72). In light of studies that show PCBP1, and several of the other DDX and DHX interactors we have identified as being PARP1 interactors (Fig. 6*C*) (73, 74), we hypothesize that the increased mutational burden we observe in PCBP1-depleted cells coinciding with basal DNA damage at PCBP1 binding sites (6) may be due to improper recruitment of DDX and DHX family proteins. We are particularly interested in the PCBP1-PARP1 axis as PARP inhibitors are extensively used therapeutically to treat several types of cancer, primarily in patients with DNA repair pathway deficiencies (75). Therefore, a deeper understanding of PCBP1’s role in DNA damage and whether it involves its PARP1 and helicase interactions will have clinical implications.

Our work contributes to the multifaceted roles of PCBP1 and its importance in cancer biology. PCBP1 is known to regulate alternative splicing (9, 76) and a recently published study found that G4 structures are commonly found at the 3′-ends of splice sites (77). This may explain the abundance of intronic sites in the PCBP1 ChIP-seq. Both datasets show that genes exhibiting PCBP1-bound promoters have a high likelihood of containing PCBP1 binding sites throughout the gene body (Fig. S1*A*). We chose to study *CDKN1A* as it is a gene that contains PCBP1 DNA binding sites throughout its structure as identified by ChIP analyses. By focusing on the PCBP1 binding site closest to the TSS, we found a function for PCBP1 in suppressing RNA polymerase II’s activity which we hypothesized is enhanced by a promoter G4. Additionally, we have identified both human and mouse cell lines that exhibit increased *CDKN1A* expression (Figs. 2*E* and S2). This adds to previous studies describing PCBP1’s post-transcriptional control of *CDKN1A* expression by regulating its mRNA stability and translation (78). Most importantly, we’ve shown for the first time that PCBP1’s regulation of *CDKN1A* starts upstream of the TSS, where it is regulated in part through a promoter G4 sequence, R-loop, and DHX9 recruitment. In light of our recent work showing that PCBP1 depletion leads to genome instability (6), which under normal circumstances would lead to the induction of p53 and transcription of *CDKN1A* (79), we expanded our search for genes that are regulated by PCBP1 on the transcriptional level to several of the differentially expressed genes that exhibited high fold changes and had PCBP1 promoter binding sequences (Fig. 2*E*). Strikingly PCBP1 depletion leads to a loss of DHX9 and an increase in G4s and R-loops in both up and downregulated differentially expressed genes.

In the context of our studies on TGFb-mediated EMT and metastasis (7, 10, 12, 29, 80), the interaction of PCBP1 with DHX9 and R-loops is of particular interest. DHX9 has been reported to inhibit EMT in A549 lung adenocarcinoma cells, is overexpressed in several cancer types, including lung, breast, and colorectal cancer, and is implicated in the TGF*β* pathway (47, 81). Also, in patients with amyotrophic lateral sclerosis 4 (ALS4), caused by mutations in senataxin, R-loops have been shown to block DNA methylation at gene promoters and inhibit DNA methyl-transferase-1 (DNMT1) activity leading to activation of the TGFb pathway (82). As we have shown that cancer cell signaling can affect the function, localization, and action of PCBP1 in the TGFb and metastatic cascades, understanding its nuclear function and whether it plays a role in transcribing pro-metastatic and pro-tumorigenic genes by regulating G4s and R-loop levels is important and has clinical implications. Our future studies will therefore focus on the transcriptional regulation of pro-metastatic genes that require epistatic regulation through both PCBP1/DHX9 interactions.

## Experimental procedures

### Cell culture and reagents

A549, NMuMG, HMLE, and HeLa S3 cells were obtained from the American Type Culture Collection (ATCC). A549, NMuMG, and HMLE derivative cell lines stably expressing control and PCBP1 shRNAs were cultured in the presence of puromycin. HeLa cell lines overexpressing V5-RNAseH were prepared as described in the R-ChIP protocol and were maintained in media containing Hygromycin (55). All cells were cultured in Dulbecco’s modified Eagle’s medium (DMEM, cat. No. SH30081.01; GE Healthcare) supplemented with 10% fetal bovine serum, 1% antibiotic/antimycotic solution, and prophylactic Plasmoscin *mycoplasma* elimination reagent. Cells were maintained at 37 °C and 5% CO_2_. Puromycin and Hygromycin were purchased from InvivoGen. Oligonucleotides used in this study are found in Table S3.

### Antibodies

Antibodies used for western blotting, immunofluorescence, and chromatin immunoprecipitation were against the following proteins. Anti-PCBP1 (RN024P) was purchased from MBL Technologies. Anti-PCBP2 (83017S), anti-RBP1-CTD (2629S), anti-RBP1-NTD, mouse anti-IgG conformation-specific (3678), anti-beta-Actin (4967) and anti-DDX21 (75,762) were purchased from Cell Signaling Technologies. Anti-DHX9 (ab26271& ab183731-used for ChIP) were purchased from Abcam. Anti-DHX36 (525-A), anti-DHX5 (523-A), and anti-DDX6 (460-A) were purchased from Bethyl Laboratories. Anti-p21 antibody (556,431) was purchased from BD Biosciences. Anti-V5 (R960-25) and anti-HSP90 (C45G5) were purchased from Thermo Fisher. Anti-G-quadruplex BG4 antibody (MABE917) was purchased from Sigma-Aldrich. All primary antibodies were diluted in 5% bovine serum albumin (BSA) for western blotting.

### Electromobility shift assay (EMSA)

Recombinant GST-PCBP1 protein was prepared and EMSAs were performed as described previously (6). 50 pmol DNA was 5′-end labeled in a 50 μl reaction containing T4 polynucleotide kinase (PNK) buffer (New England Biolabs), 150 μCi *γ*-^32^P ATP, and T4 PNK (10 U/μl, NEB 37 °C for 1 h). The labeled DNA was then purified using Sephadex G25 (GE Healthcare) spin columns. ^32^P-labeled oligodeoxynucleotides (20 fmol) were mixed with various concentrations of GST-PCBP1 in 50 μl reaction buffer that contained 25 mM Tris–HCl (pH 7.6), 1 mM EDTA, 2% glycerol, 10 mM *β*ME, 50 μg/ml BSA on ice. The reaction mix was then incubated at RT for 15 min before being mixed with loading dye containing 5% glycerol and bromophenol blue. The samples were loaded on 10% polyacrylamide gels run at 150 V for 90 min in 1× Tris-Borate EDTE (TBE) running buffer. The gels were exposed to a phosphoimager screen and analyzed by a Typhoon FLA 1900 phosphoimager, bands were analyzed and quantified using ImageJ.

### Immunoblotting

Immunoblot analysis was performed as described previously (6). Briefly, all cell extracts were fractionated by SDS-PAGE and transferred to PVDF membranes (Bio-Rad). Proteins were probed with primary antibodies (at dilutions ranging from 1:500 to 1:5000) and then with appropriate secondary antibody-HRP conjugates (1:5000 dilutions). Western blots were developed using ECL and imaged using a ChemiDoc Gel Imaging System (Bio-Rad).

### Luciferase assay

Cells (5 × 10^5^) were plated on 24-well plates, after 24 h (70% confluence), cells were cotransfected with 500 ng pGL2-p21 promoter-Luc (Addgene #33021) and 50 ng of *Renilla* luciferase reporter plasmid using the Lipofectamine 3000 transfection reagent (Thermo Fisher) according to manufacturer’s instructions. Luciferase production was assayed using the dual-luciferase reporter assay system (Promega). Luciferase activity was normalized by *Renilla* expression. The results were expressed as fold induction of luciferase after normalization.

### CRISPR knockout of PCBP1

To knockout *PCBP1* in A549 and HeLa cells, 100K cells were transfected with multiplex sgRNAs (Synthego) targeting human *PCBP1* (CCGCCGCTCGCCATGGATGC, TCGGCTTCTTATGCACGGAA, and GCGCGGATCAACATCTCGGA) and Cas9 nuclease using the Lipofectamine CRISPRMAX Transfection Reagent (Thermo Fisher) according to the Synthego CRISPR editing protocol. 72 h post-transfection, cells were divided and either collected for knockout analysis or transferred to 96-well plates for single clonal expansion. *PCBP1* knockout was assessed by sequencing genomic DNA from knockout pools and post-clonally selected cells after PCR amplification using forward primer GCCAAAGACTTGACCACGTAA and reverse primer CAGGCAAATCTGCTTGACAC, spanning the Cas9-sgRNA cleavage site. PCR amplicons were then Sanger-sequenced to identify the knockout efficiency using Inference of CRISPR Edits (ICE) from Synthego.

### Immunofluorescence

For immunofluorescence, cells were fixed by adding paraformaldehyde directly into media to a concentration of 1% paraformaldehyde, were washed once in ice-cold PBS, followed by a 30 min incubation in PBS containing 2% paraformaldehyde. Cells were then washed and permeabilized with 0.1% Triton X-100 for 30 min at 4 °C. Cells were then incubated overnight for blocking in PBS containing 2% BSA and 0.1% Triton X-100, or SuperBlock (Thermo Fisher) containing 0.1% Triton X-100. The following day, the primary antibody was incubated in SuperBlock containing 0.05% (wt/vol) Triton X-100 for 4 h at 4 °C. Cells were then washed thrice with PBS containing 0.1% Tween-20 (PBS-T), and secondary antibodies conjugated with Alexa Flour (Life Technologies) were added in SuperBlock for 1 h at room temperature, followed by three washes with PBS-T. Cells were mounted using DAPI Flouromount G (SouthernBiotech) and imaged using an FV10i confocal laser scanning microscope (Olympus).

### RNA-sequencing and analysis

Total RNA was isolated using TRIzol (Thermo Fisher Scientific). RNA integrity was assessed using a Bioanalyzer (Agilent 2100). 850 μg of total RNA was used to construct libraries with the New England Biolabs NEBNext rRNA Depletion Kit (Cat# E6310X) and Ultra II Directional RNA Library Prep Kit for Illumina (Cat# 7760L) according to the manufacturer’s instructions. Dual-indexed libraries were pooled and sequenced at VANTAGE (Vanderbilt University Medical Center) on an Illumina NovaSeq 6000 (S4 flow cell) to a depth of approximately 50 million paired-end 150 bp reads per library. For RNA-seq analysis, the reads were aligned to the GRChg38 human genome assembly with Bowtie2 and were quantified to the reference genome using Partek (E/M). Differential expression analysis was performed in Partek Flow using the DEseq2.

### PCR analysis

For gene expression analysis by PCR, cDNA was synthesized from 1000 ng of total RNA using qScript cDNA synthesis kits (Quantabio). After qScript cDNA synthesis, cDNA was diluted two-fold, and 0.5 μl of cDNA was used per PCR reaction. For ChIP-qPCR samples, 1 μl of purified DNA was used per 10 μl reaction. Semi-quantitative PCR was conducted using Maxima Hot Start PCR Master Mix (Thermo Fisher Scientific). Real-time quantitative PCR was conducted using iQ SYBR Green Supermix (Bio-Rad) using CFX384 Real-Time System (BioRad). Relative gene expression was calculated using RFX Manager software, and genes were normalized to either *GAPDH* or *ACTB* as internal controls.

### Co-immunoprecipitation (co-IP)

Cells were lysed in IP lysis buffer (20 mM Tris-HCl pH 7.4, 150 mM NaCl, 1 mM EDTA, 0.5% NP-40, 1% Triton X-100, 1× protease/phosphatase inhibitor cocktail) and incubated on ice for 30 min before centrifugation at 20,000*g* for 15 min. Protein estimation of cleared cell lysates was performed by Bradford assay. 1 mg of lysates per primary antibody were measured, brought to a volume of 1 ml using HBST buffer (20 mM Hepes pH 7.4, 150 mM NaCl, 0.05% Triton X-100), and precleared at room temperature for 1 h using 25 μl of Protein A Sepharose 4B bead slurry (Thermo Fisher). After preclearing, primary antibodies were added overnight at 4 °C with rotation. Lysate:antibody solution was then incubated with protein A Sepharose 4B beads for 2 h at 4 °C with rotation. Beads were washed 4 times in HBST, then twice with HBS (20 mM Hepes ph7.4, 150 mM NaCl), the supernatant was removed, and 30 μl of 2× Laemmli buffer was added. Proteins were eluted by heat denaturation at 95 °C for 5 min.

### Mass spectrometry

V5-PCBP1 bound proteins were isolated by immunoprecipitation as described above. Next, they were resolved briefly by SDS-PAGE, stained with Coomassie Brilliant Blue, and then excised. The gel fragments were sent to the Taplin Mass Spectrometry Facility (Harvard Medical School) for processing and analysis. The average sum intensity of proteins isolated above the background and the number of unique peptides corresponding to identified proteins was determined for each condition and quantified for enrichment over mock-transfected cells. Mass spectrometry data is included in Table S2.

### Chromatin immunoprecipitation (ChIP)

For PCBP1, DHX9, and V5 ChIP experiments, cells were grown to 70 to 80% confluency in 150 mM cell culture dishes, formaldehyde was added directly into media to a final concentration of 1%, and dishes were rocked at 30 RPM, room temperature for 10 min. Freshly prepared glycine solution was added to a concentration of 125 mM and rocked for 20 min. Cells were then washed twice in ice-cold PBS, then scraped, and collected in ice-cold PBS. Cells were then pelleted at 500*g* for 3 min and pellets were resuspended and brought to a volume of 1 ml in ChIP lysis buffer (150 mM NaCl, 1 mM EDTA, 25 mM Tris-HCl (ph7.6) (Thermo Fisher), 0.5% Triton X-100 (wt/vol), 0.5% NP-40, 0.1% SDS, and 0.1% sodium deoxycholate). Crosslinked cell lysates were frozen at −80 °C, then thawed on ice prior to sonication to facilitate lysis. Cell lysates were then transferred to 1 ml Millitubes (Covaris 520,128) and sonicated using a Covaris S220 Focused-Ultrasonicator using settings (duty cycle = 5, PIP = 140, cycles/burst = 200, time = 25 min). After sonication, lysates were centrifuged for 20 min at 20,000*g*. 50 μl (1/20th) of cleared lysates were removed for DNA purification by phenol:chloroform extraction, the remaining cleared lysate was divided equally into two equal volumes and brought to a volume of 1.5 ml using ChIP lysis buffer without SDS, primary antibodies were added and samples were rotated overnight at 4 °C. Next, protein A protein A Sepharose 4B beads were equilibrated in lysis buffer (without SDS), and 50 μl of slurry was added to each sample and rotated for two hours at 4 °C. Next, samples were centrifuged at 1000*g* for 3 min, and beads were watched three times in lysis buffer (without SDS), twice in lysis buffer containing no detergents, and twice in elution buffer. After the final wash, beads were resuspended in 100 μl of TE. Input samples and beads in TE were then kept at 65 °C overnight to reverse crosslinking. The following day, samples were treated with RNAse A at 20 μg/ml (NEB T3010) for one hour at 37 °C, then brought SDS was added to a concentration of 0.2% and samples were treated with Proteinase K at a final concentration of 20 μg/ml for 1 h at 37 °C. DNA was then extracted using a standard phenol: chloroform extraction. For BG4 ChIP, we referenced a previously established protocol (36), and performed preparation of crosslinked lysates and sonication as described above. We used 25 μg of chromatin per reaction and 10 μl (0.25 mg/ml) of BG4 antibody or Anti-Flag antibody for control reactions, 100 μl of Pierce Anti-DYDDDDK magnetic beads (Thermo Fisher) per reaction.

### R-loop dot blot

R-loop dot blots were performed using an established protocol (83). Briefly, cells were harvested, counted, and 2 × 10^6^ cells were pelleted per sample. Cell lysis buffer (0.5% NP-40, 80 mM KCl, 5 mM PIPES ph8.0). Nuclear lysis buffer (1% SDS, 35 mM Tris-HCl (pH 8.0), 5 mM EDTA). Elution buffer (10 mM Tris-Cl, pH 8.5). Nuclei were sonicated 6 × 20 s at 40% power on a Branson SFX150 Sonicator. Genomic DNA containing RNA-DNA hybrids was purified using phenol-chloroform extraction, samples were then diluted, and 10 μg of DNA was either treated with either RNAse H (5 U/μl, NEB M0523) or mock (elution buffer) for 15 min at 37 °C. Samples were loaded onto Zeta-Probe (Bio-Rad) blotting membrane using a dot-blot apparatus (Bio-Rad), and membranes were crosslinked using the auto-crosslink setting. Membranes were blocked in blocking solution (5% BSA in TBST) for 1 h at room temperature. Primary antibodies, either anti-S9.6 (Kerafast ENH001, 1:1000 dilution) or anti-dsDNA (Abcam ab27156, 1:5000 dilution), were added in 5% BSA in TBST, and membranes were incubated with rotation overnight at 4 °C.

### Statistical analysis

All data are presented as means ± SEM. Comparisons between groups were analyzed by unpaired two-tailed Student’s *t* test or one-way/two-way analysis of variance (ANOVA). Statistical analysis was performed using GraphPad Prism 8. *p* < 0.05 was considered statistically significant (∗*p* < 0.05, ∗∗*p* < 0.01, ∗∗∗*p* < 0.001, and ∗∗∗∗*p* < 0.0001; ns, not statistically significant).

## Data availability

All data needed to evaluate the conclusions in the paper are present in the paper and/or the Supplementary Materials. The RNA-seq and other raw sequencing data and files used for data analysis have been deposited in the Gene Expression Omnibus under accession number GSE225637. ENCODE consortium (84) datasets used in our analysis include ChIP-seq datasets GSE174944 (K562) and GSE106035 (HepG2), and RNA-seq datasets GSE80899 (K562) and GSE80915 (HepG2). NMuMG shPCBP1 RNA sequencing data are accessible through GEO Series accession number GSE146273.

## Supporting information

Supplementary Data are available at JBC online. This article contains supporting information.

## Conflict of interest

The authors declare that they have no conflicts of interest with the contents of this article.
