# Supplementary material for: The RNA-binding protein PCBP1 modulates transcription by recruiting the G-quadruplex-specific helicase DHX9
Source: J Biol Chem. 2024 Sep 27;300(11):107830. doi: 10.1016/j.jbc.2024.107830 (PMC11538862; doi:10.1016/j.jbc.2024.107830)
Supplement: Supporting Information [file mmc1.docx]

Supporting Information for

**The RNA binding protein PCBP1 modulates transcription by recruiting the G-quadruplex-specific helicase DHX9**

Joseph A.Q. Karam *et al.*

**Table of Contents**

Figs. S1 to S4

Legends for Tables S1 to S3

**Other Supplementary Materials for this manuscript include the following:**

Tables S1 to S3 (Excel Files)


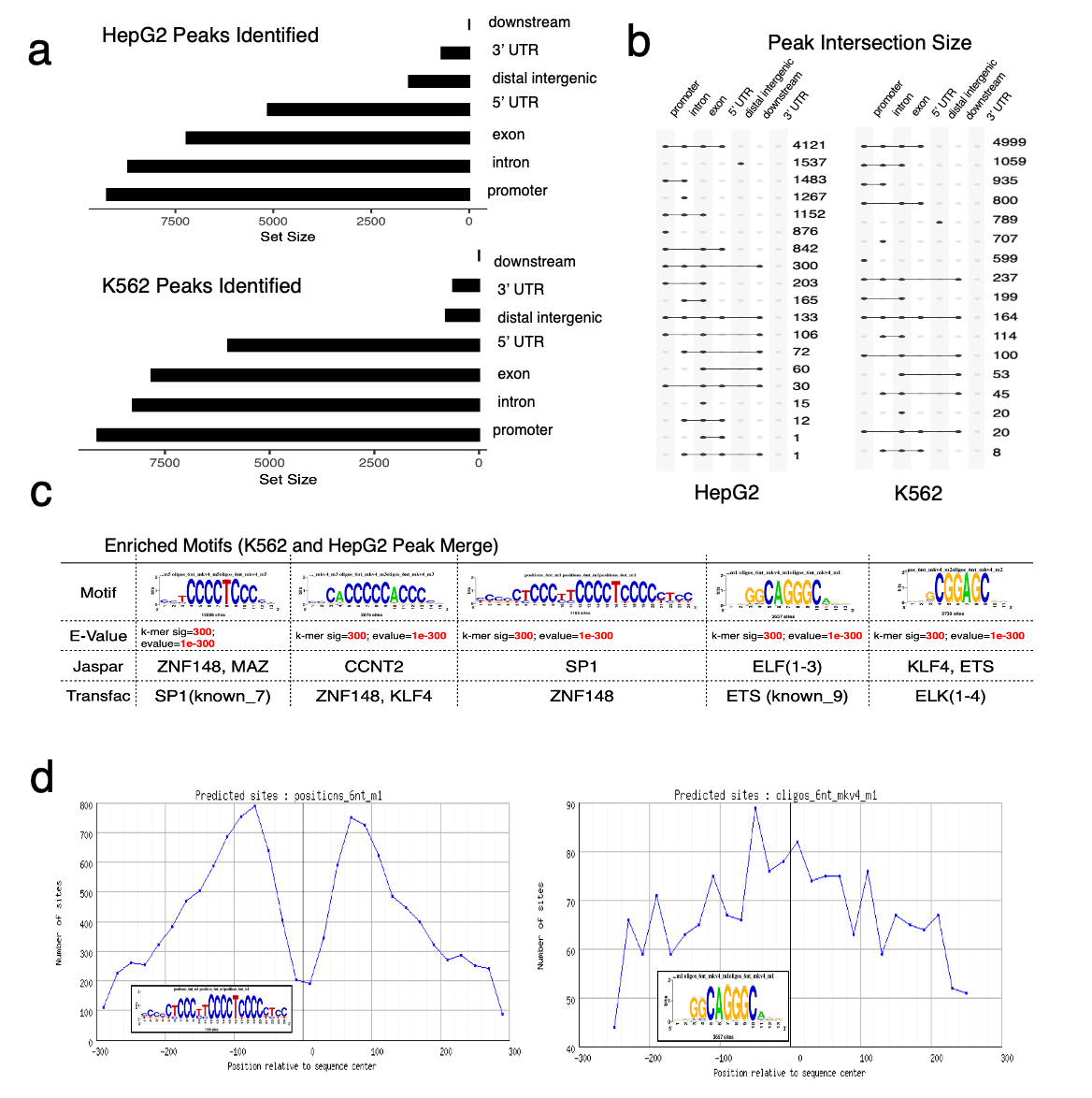


Fig. S1. Analysis of PCBP1 chIP-seq binding sites. (A) Peak set sizes from chIPseeker for PCBP1 chIP-seq in HepG2 and K562 cell lines. (B) Peak intersection showing number of genes with PCBP1 binding sites that overlap multiple features. (C) Motifs enriched using RSAT peak motifs on chIP-seq peaks found in both K562 and HepG2 PCBP1 chIP-seq datasets (n=4,253 sites). (D) RSAT peak motifs chIP-seq distribution plots of the polycytosine PCBP1 consensus motif (left) and an enriched KLF4/ETS motif (right).


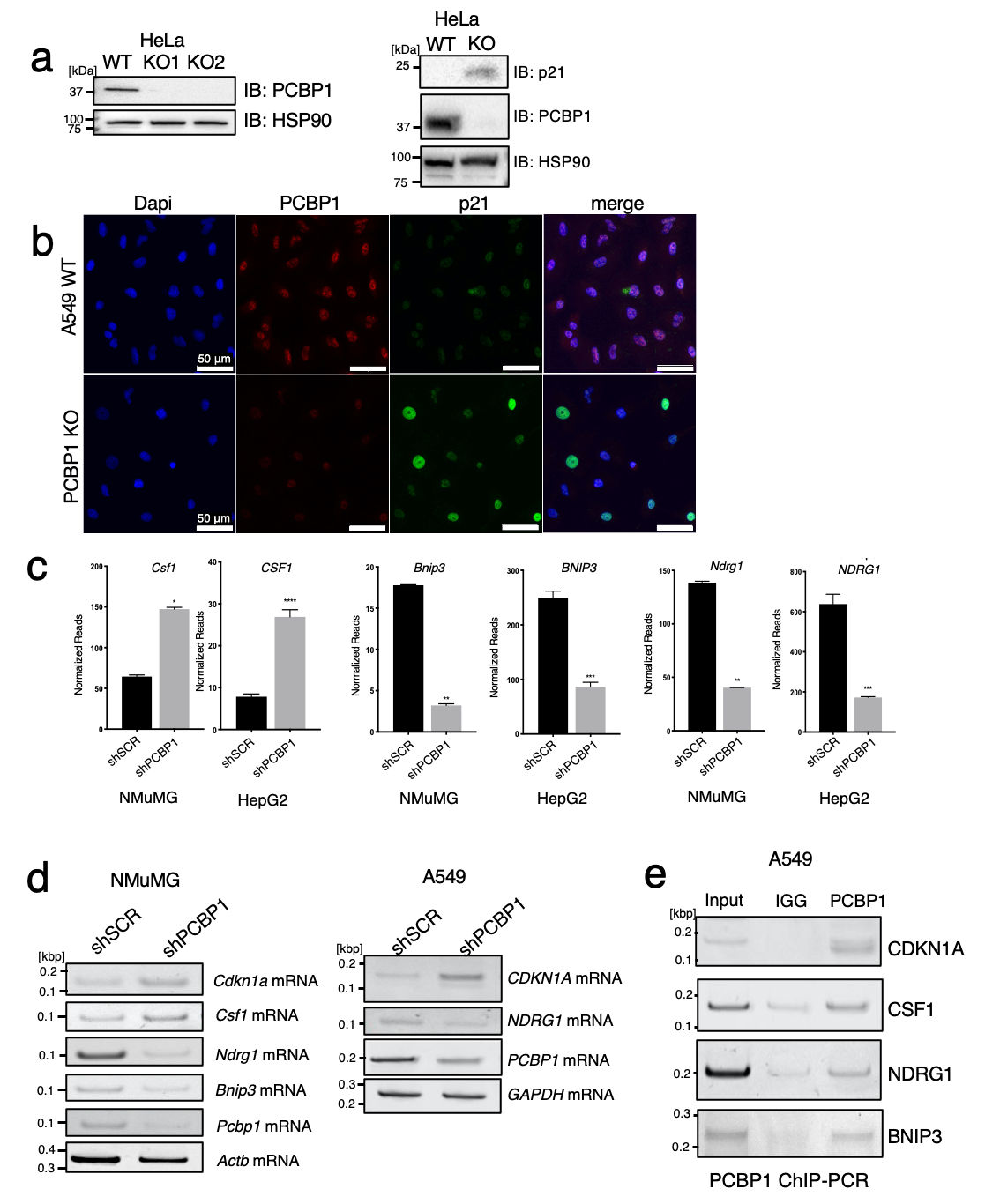


Fig. S2. Conserved binding of PCBP1 to regulate transcription. (A) Immunoblots of CRISPR HeLa PCBP1 knockout cell lines (left), and of p21 upregulation in HeLa PCBP1 knockout cells relative to wildtype HeLa cells (right). (B) Immunofluorescence of PCBP1 and p21 in A549 WT and PCBP1 knockout cells, nuclei are stained with dapi. (C) Normalized reads from RNA-seq experiments comparing *CSF1, BNIP3,* and *NDRG1* levels in NMuMG and HepG2 cells stably expressing control or PCBP1 targeting shRNAs. (D) End-point PCR showing levels of *Cdkn1a, Csf1, Ndrg1, and Bnip3* in NMuMG cells expressing control or PCBP1 targeting shRNAs (left). End-point PCR showing levels of *CDKN1A* and *NDRG1* in A549 cells expressing control or PCBP1 targeting shRNAs. (E). PCBP1 chIP followed by endpoint PCR targeting the promoters of *CDKN1A, CSF1, NDRG1,* and *BNIP3* to test PCBP1 enrichment.


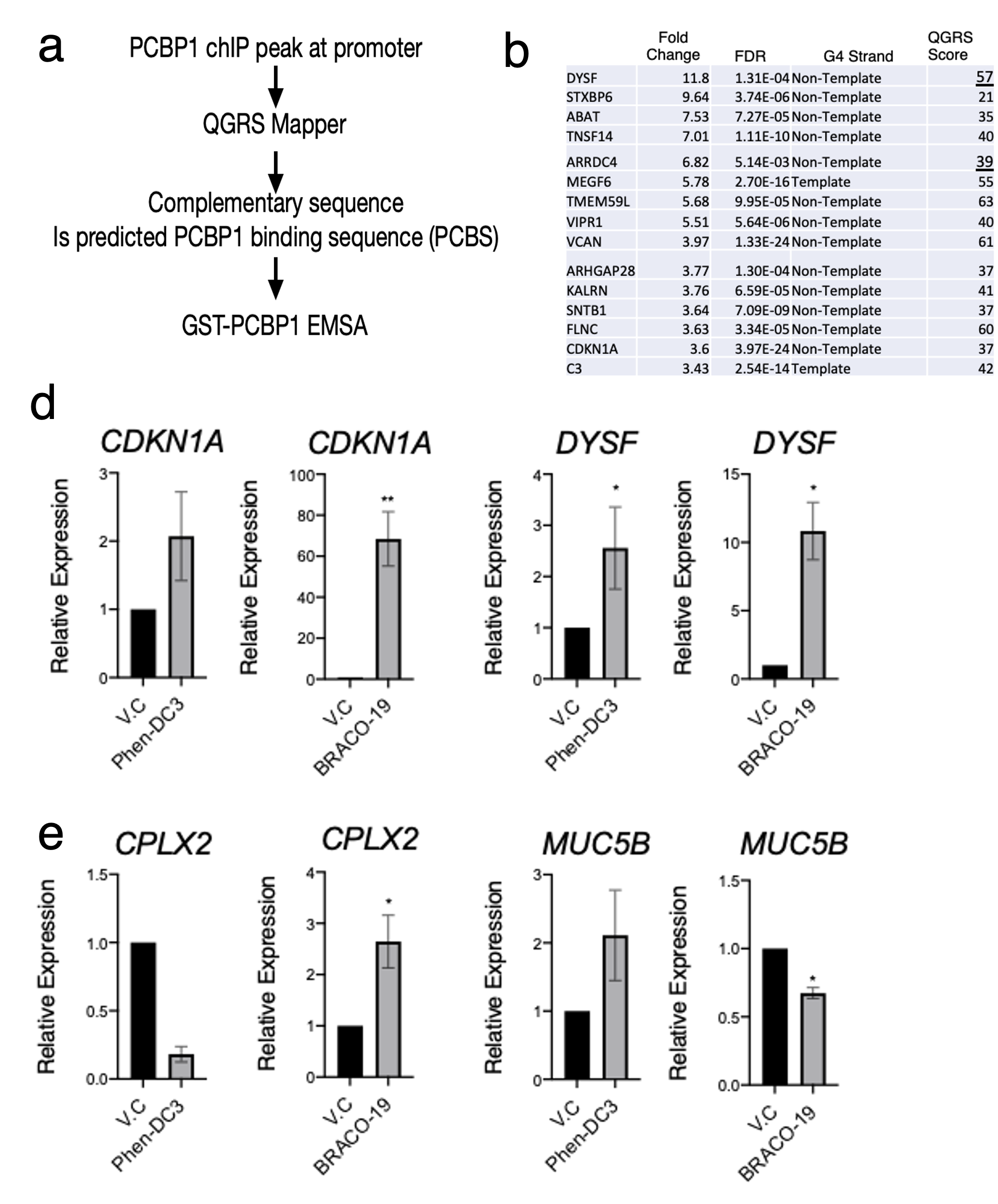


Fig. S3. Identification of G4s at PCBP1 binding sites. (A) Schematic of our workflow to identify G4s in PCBP1 bound promoters. After identifying PCBP1 bound promoters, we used QGRS mapper on the promoter DNA and its revere complement to identify the highest scoring G4 within the PCBP1 bound region. We used the highest scoring G4 and its complementary polycytosine site to perform EMSAs using GST-PCBP1. (B) Highest scoring G4 sequence strand location in most upregulated genes from A549 PCBP1 KO RNA-seq. Fold change and FDR from Deseq2 and promoter QGRS G4 score shown. (C) Control for BG4 chIP experiments, testing enrichment of loci not predicted to form G4 structures in *DYSF, VCAN, MUC5B*, and *VCAN*. (D) Testing the effect of Phen-DC3 and BRACO-19 treatment on the transcription of genes upregulated in PCBP1 KO cells due to loss of promoter bound PCBP1. (E) Testing the effect of Phen-DC3 and BRACO-19 treatment on the transcription of genes downregulated in PCBP1 KO cells due to loss of promoter bound PCBP1.

a

100

75

37

[kDa]

37


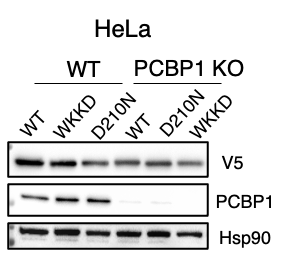


**Fig. S4.** PCBP1 and R-loops (A) Immunoblot of HeLa WT and PCBP1 knockout cells expressing V5-RNAseH1-WT, -WKKD, and -D210N

**Legends for Tables S1-S3**

**Table S1 –** Peaks identified in HepG2 and K562 chIP-sequencing datasets and the merged peaks found in both used for our analysis.

**Table S2 –** V5-PCBP1 Mass spectrometry Data.

**Table S3** – Oligonucleotides used in our study
